# Immune cell populations differ in patients undergoing revision total knee arthroplasty for arthrofibrosis

**DOI:** 10.1038/s41598-022-22175-3

**Published:** 2022-12-31

**Authors:** Afton K. Limberg, Christopher G. Salib, Meagan E. Tibbo, Juan S. Vargas-Hernandez, Jacob W. Bettencourt, Banu Bayram, Charlotte E. Berry, Amel Dudakovic, Brad Bolon, Andre J. van Wijnen, Mark E. Morrey, Joaquin Sanchez-Sotelo, Daniel J. Berry, Jodi M. Carter, Matthew P. Abdel

**Affiliations:** 1 Department of Orthopedic Surgery, Mayo Clinic, 200 First Street S.W, Rochester, MN 55905 USA; 2 GEMpath Inc, 1927 Lincoln Street, Longmount, CO 80501 USA; 3 Department of Biochemistry, University of Vermont, 89 Beaumont Avenue, Burlington, VT 05405 USA; 4 Department of Laboratory Medicine and Pathology, Mayo Clinic, 200 First Street S.W, Rochester, MN 55905 USA

**Keywords:** Bone, Cell biology

## Abstract

Arthrofibrosis following total knee arthroplasty (TKA) is a debilitating condition typically diagnosed based on clinical findings. To gain insight into the histopathologic immune cell microenvironment of arthrofibrosis, we assessed the extent of tissue fibrosis and quantified immune cell populations in specific tissue regions of the posterior capsule. We investigated specimens from three prospectively-collected, matched cohorts, grouped as patients receiving a primary TKA for osteoarthritis, revision TKA for arthrofibrosis, and revision TKA for non-arthrofibrotic, non-infectious reasons. Specimens were evaluated using hematoxylin and eosin staining, picrosirius red staining, immunofluorescence, and immunohistochemistry with Aperio®-based digital image analysis. Increased collagen deposition and increased number of α-SMA/ACTA2 expressing myofibroblasts were present in the arthrofibrosis group compared to the two non-arthrofibrotic groups. CD163 + macrophages were the most abundant immune cell type in any capsular sample with specific enrichment in the synovial tissue. CD163 + macrophages were significantly decreased in the fibrotic tissue region of arthrofibrosis patients compared to the patients with primary TKA, and significantly increased in adipose tissue region of arthrofibrotic specimens compared to non-arthrofibrotic specimens. Synovial CD117 + mast cells were significantly decreased in arthrofibrotic adipose tissue. Together, these findings inform diagnostic and targeted therapeutic strategies by providing insight into the underlying pathogenetic mechanisms of arthrofibrosis.

## Introduction

Acquired idiopathic stiffness is a common postoperative complication following total knee arthroplasty (TKA), occurring in approximately 4% of all TKA patients^1^. A subset of these patients have arthrofibrosis, defined by a pro-inflammatory response leading to excessively dense scarring in peri-articular soft tissue resulting in a restricted range of motion. Unfortunately, the limited motion also compromises functional outcomes. Moreover, limited treatment options exist^2,3^. Current treatment strategies including physical therapy, manipulation under anesthesia (MUA), arthroscopic or open lysis of adhesions, and/or revision TKA, provide limited success^2,4–7^.

The variable success rate with current treatment options may arise in part from lack of standardized characterization, definition, and description of arthrofibrosis. At present, the diagnosis of arthrofibrosis is based solely on clinical evaluation and the judgement of the treating surgeon. As such, arthrofibrosis is often described using clinical parameters such as range of motion (ROM), degrees of flexion and or extension contracture, and several different patient-reported pain and functional scores^7^. This subjective diagnostic approach has led to varying definitions and reported outcomes for this condition^2^.

The subjectivity in diagnosis may, in part, be due to poorly understood cellular mechanisms driving arthrofibrosis and the lack of a comprehensive pathologic characterization of the tissue and related diagnostic tests. Several potential mechanisms have been proposed many involving increased myofibroblast activity as measured by enhanced expression of α-smooth muscle actin (α-SMA/ACTA2)^8–10^, a characteristic of fibrotic tissue. In fibrosis of other organ tissue types, cytokines produced by mast cells and macrophages have been shown to play a major role in the development of dense fibrotic tissue^11–13^. Macrophages have diverse and adaptive phenotypes with both pro-inflammatory and anti-inflammatory as well as pro-fibrotic and anti-fibrotic roles, respectively, and are associated with wound healing^14,15^. In particular, CD163 + macrophages have been shown to be key inflammatory cells involved with rheumatoid arthritis^16,17^. Type 2 immunity-related cells, and specifically mast cells, have also been shown to regulate tissue repair and regeneration^18^. Likewise, emerging studies have demonstrated an association between CD117 + mast cells and arthrofibrosis^19,20^. These studies have shown increased mast cell numbers in a rabbit model of arthrofibrosis, as well as elevated serum mast cell tryptase levels. However, the relevance and clinical utility of manipulating these cell populations in arthrofibrosis remain poorly defined.

This study aims to gain insight into the pathobiology of arthrofibrosis by comprehensive assessment of tissue fibrosis in association with quantification of immune cells in knee tissues. We quantified α-SMA/ACTA2 + myofibroblasts, CD163 + macrophages and CD117 + mast cells in posterior capsule specimens from three prospectively-collected, matched patient cohorts: primary TKAs, revision TKAs for arthrofibrosis, and revision TKAs for non-arthrofibrotic reasons. The novelty of this study includes the matched patient groups with strict inclusion criteria, examining the above specified immune cell markers in the different regions of the tissue specimens (fibrous, adipose and synovial areas), the correlation of these histopathologic results with clinical outcomes, and comparing the immune cell data with gene expression analysis. The main result is that these specimens exhibit differences in the distribution and quantity of cells with these biomarkers that reflect their clinical status.

## Patients and methods

### Patient enrollment

Prior to the study, all patients provided informed consent according to a protocol approved by the Institutional Review Board (IRB) protocol (09–000115, Mayo Clinic, Rochester, MN). All methods were carried out in accordance with the relevant guidelines and regulations of this protocol. Patients were matched according to age (± 5 years), sex, body mass index (BMI; ± 5 kg/m^2^), smoking history and diabetes status, because these patient parameters represent independent risk factors for arthrofibrosis^3^. Patient-reported outcomes were calculated preoperatively and at the most recent follow-up appointment using Knee Society scores (KSSs)^21^.

A total of 35 patient samples were collected: 14 from primary TKAs, 14 from revision TKAs for arthrofibrosis, and 7 from revision TKAs for aseptic, non-fibrotic conditions (Table 1). The first samples collected where those from the arthrofibrosis group, as this is the rarest of the three patient populations. The primary TKA and revision TKA for non-arthrofibrotic conditions served as control groups and were matched to the arthrofibrosis samples. With the strict inclusion and matching criteria, it was difficult to match the revision TKA for non-arthrofibrotic to the revision arthrofibrotic group, which is why this group has fewer samples. The mean time interval from primary TKA to revision TKA in the revision for arthrofibrosis group was 3 years (range, 1–10 years). The mean interval time from primary TKA to revision in the revision TKA for non-arthrofibrotic reasons group was 7 years (range, 4–11 years) (Table 1).

**Table 1:** Patient demographics for each group including primary TKA (PTKA), revision TKA for arthrofibrosis (RTKA-A) and revision TKA for non-arthrofibrotic etiologies (RTKA-NA).

Group	Number of patients	Mean age, years (SD)	Sex	Mean BMI, kg/m^3^ (SD)	Smokers	Diabetics	Mean time to RTKA, years	Mean preoperative ROM
Primary TKA (PTKA)	14	65 (9)	5 Female	34 (5)	14% (n = 2)	7% (n = 1)	–	109°
			9 Male					
Arthrofibrotic (RTKA-A)	14	65 (10)	6 Female	34 (7)	21% (n = 3)	14% (n = 2)	3	68°
			8 Male					
Non-Arthrofibrotic (RTKA-NA)	7	67 (7)	4 Female	30 (5)	0% (n = 0)	0% (n = 0)	7	110°
			3 Male					

Age is represented in years at time of surgery.

*BMI* = body mass index, *ROM* = range of motion.

### Tissue acquisition and handling

**Figure 1:**
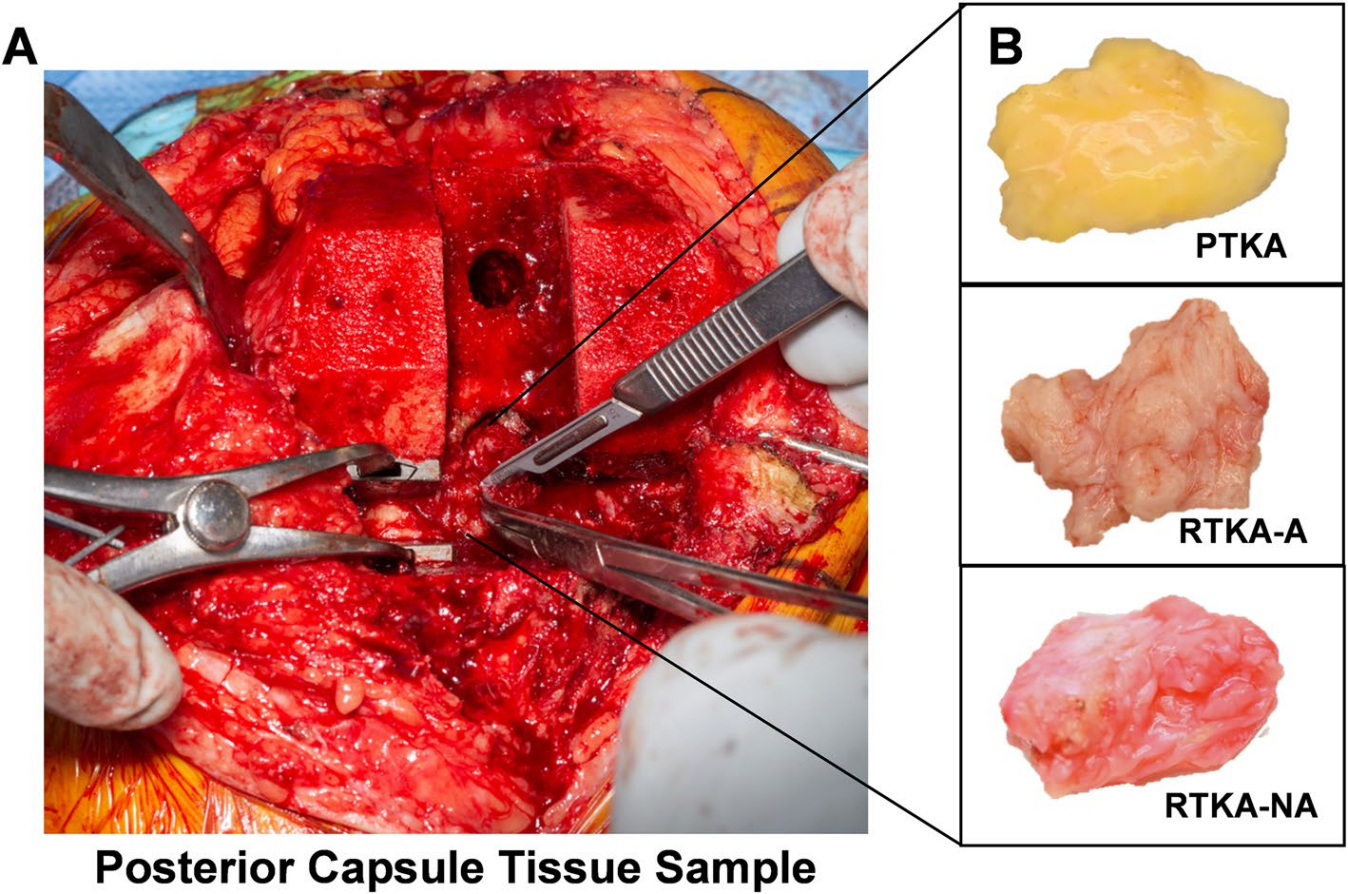
Intraoperative harvesting of posterior capsule tissue samples. An intraoperative photograph of the posterior capsule tissue sampling site (**A**). Gross appearance of representative posterior capsule specimens from each of the three patient groups (**B**). Abbreviations: *TKA* = total knee arthroplasty, *PTKA* = primary TKA, *RTKA-A* = revision TKA for arthrofibrosis, *RTKA-NA* = revision TKA for non-arthrofibrotic conditions.

At the time of surgery, posterior capsular tissues were harvested intraoperatively under sterile conditions upon visual verification of the anatomical provenance of the sample by the operating surgeons (Fig. 1). Samples were fixed by immersion in neutral-buffered 10% formalin for 48 h at room temperature (RT), and processed into paraffin per standard histology protocols.

Tissue sectioning and immunohistochemistry (IHC) staining were performed at our Pathology Research Core (Mayo Clinic, Rochester, MN) using the Leica Bond RX stainer (Leica, Buffalo, IL). Formalin-fixed, paraffin-embedded (FFPE) tissues were sectioned at 5 microns. Following slide preparation, IHC staining was performed for selected cell type-specific biomarkers according to the stainer manufacturer’s instructions; markers in the staining battery were selected to detect CD20 + (gene symbol MS4A1) B-lymphocytes and CD3 + T-lymphocytes, CD163 + macrophages, and CD117 + mast cells (c-Kit/KIT). Briefly, sections were dewaxed using Bond Dewax (Leica), and antigen retrieval was performed for 20 min at RT using Epitope Retrieval 2 (EDTA; Leica). The primary antibodies were diluted in Bond Diluent (Leica). Primary antibody dilutions and incubation times are listed in Supplementary Table 1. Secondary antibodies were applied for 30 min at RT. Antibody binding was demonstrated using 3, 3’-diaminobenzidene (DAB) as the chromagen.

### Histopathologic evaluation

Tissue sections were stained with conventional H&E and evaluated using a Nikon E600 bright-field light microscope by an ACVP board-certified veterinary anatomic pathologist (BBo). Fibrosis was scored using tiered, semi-quantitative scales with the following grades: within normal limits (score = 1), mild (2), moderate (3), or severe (4) lesions (Table 2)^22,23^. The criteria for H&E-stained sections were based on the extent of fibrosis. These criteria were established by an initial informed (“non-blind”) histopathologic evaluation, after which the definitive data set was produced by a coded (“blind”) histopathologic assessment.

**Table 2:** Semi-quantitative histopathologic scoring criteria for the fibrous tissue on H&E stains.

Score	Meaning	Fibrous tissue
1	“Normal”	Intra-articular and peri-articular tissue (excluding ligaments) consists mainly of white fat, with small linear foci of dense connective tissue along the margins of fat pads in < 20% of section area
2	Mild	Intra-articular and peri-articular tissue (excluding ligaments) replaced by dense connective tissue in about 21 to 50% of section area
3	Moderate	Intra-articular and peri-articular tissue (excluding ligaments) replaced by dense connective tissue in about 51 to 80% of section area
4	Severe	Intra-articular and peri-articular tissue (excluding ligaments) replaced by dense connective tissue in about > 81% of section area

### Picrosirius red

One FFPE tissue section from each patient was utilized for picrosirius red staining for collagen. Briefly, the slides were treated with xylenes, degrading alcohol dilutions (100, 95, 70 and 50%), deionized water, and then rehydrated with PBS. The Picrosirius Red solution was applied to completely cover the tissue sections for 60 min (Abcam, Cambridge, U.K., cat. # ab150681). Slides were then rinsed twice with acetic acid, and twice with ethanol. Tissue sections were then mounted with Permount mounting medium (Fisher Scientific, Waltham, MA cat. # SP15-100). After 24 h, all slides were imaged at 20X magnification on a Zeiss Axio Vert.A1 microscope with a Zeiss AxioCam ICc 5 digital camera (Zeiss, Oberkochen, Germany).

### Immunofluorescence

One FFPE section from each patient was examined by immunofluorescence microscopy to identify myofibroblasts. The initial process was similar to the Picrosirius Red staining. Following rehydration with PBS, all tissue sections were blocked at room temperature for 30 min utilizing blocking buffer (Agilent, Santa Clara, CA cat. # X090930-2/X0909) and a 1% saponin solution (Millipore Sigma, Burlington, MA cat. # SAE0073-10G). Next, primary antibodies of α-SMA (ACTA2) and Laminin (encoded by LAMA gene) were applied and slides were incubated overnight at 4 °C. The next day, slides were washed with PBS and secondary antibodies for α-SMA and Laminin were applied. All slides were left covered for 1 h at room temperature. Slides were then washed with PBS and the DAPI stain was applied at room temperature for 15 min (Sigma Aldrich, St. Louis, MO, cat. # D9542). Finally, slides were washed with PBS, mounted with ProLong Gold Antifade Mountant (ThermoFisher, cat. # P10144) and images were obtained at 20X magnification on a Zeiss Axio Vert.A1 microscope with a Zeiss AxioCam ICc 5 digital camera (Zeiss). Laminin, which stains blood vessels, and α-SMA, which stains blood vessels and myofibroblasts, were employed to detect myofibroblasts^10^. Myofibroblasts were identified as having positive α-SMA and negative Laminin staining. For each specimen, three random areas were selected and the myofibroblasts were manually counted by two independent reviewers using a blinded analysis (AKL & BBa). Interobserver and intraobserver reliability calculations were performed. For statistical analyses, the averages of the two reviewers’ counts was utilized.

### Digital image analysis for immune cell quantitation

All IHC-stained slides were scanned and the digital images were annotated to define specific tissue regions (synovium, fibrous tissue and adipose tissue). For all immunofluorescence signals, digital images were assessed with optimized algorithms (Aperio ImageScope software, Leica) to quantify the density (cell/mm^2^ tissue) of IHC + cells for the entire tissue piece or within the annotated tissue regions. All slides and algorithms were reviewed by a board-certified anatomic pathologist (JMC).

### RNA-sequencing

Previously, we have characterized the mRNA transcriptome of tissues taken from primary TKAs, revision TKAs for arthrofibrosis, and revision TKAs for aseptic, non-fibrotic conditions utilizing RNA-sequencing (RNA-seq)^24^. A standard RNA-seq pipeline was employed to obtain normalized gene counts for paired-end reads, in which MAPRSeq was used to obtain expression values for each gene that normalized to 1 million reads and corrected for gene length (Fragments of reads Per Kilobase pair per Million mapped reads, FPKM). This data set has been deposited in the Gene Expression Omnibus (GEO) database (Accession # GSE135854).

### Statistics

Data are represented as mean ± standard deviation (SD). The fibrosis scoring data also includes mode values for each group. Direct comparisons of histopathologic scores among the three patient groups were performed first using a Kruskal-Wallis test, assuming nonparametric variance among groups. If a significant p-value was identified, these differences were investigated further with individual Wilcoxon-rank sum tests for the multiple comparisons. Effect size was calculated for each comparison with the arthrofibrosis group and is reported as Cohen’s *d* following each p-value^25^. All effect size values can be found in Supplementary Table 2. The possible correlation between the densities of positively stained cells and the patient demographics of age, sex and BMI were evaluated using the Spearman’s rank correlation coefficient. Association between the KSS^21^ and cell density for various cell types were also tested using the Spearman’s rank correlation. Differences occurring with < 5% likelihood of being due to chance (p < 0.05) were considered to be statistically significant. Reliability calculations were performed for the myofibroblast cell counting using interclass and intraclass correlation coefficients (ICCs)^26^. These calculations were based on determinations by two independent observers who counted the myofibroblasts on all sections, while one observer recounted all Sections 2 weeks after the initial count was completed. Both GraphPad prism version 9.0.0 (GraphPad Software; San Diego, California) and IBM SPSS Statistics 27 (IBM Statistical Software; Chicago, Illinois) were applied for all statistical analyses.

## Results

### Histopathologic scoring of fibrosis

**Figure 2:**
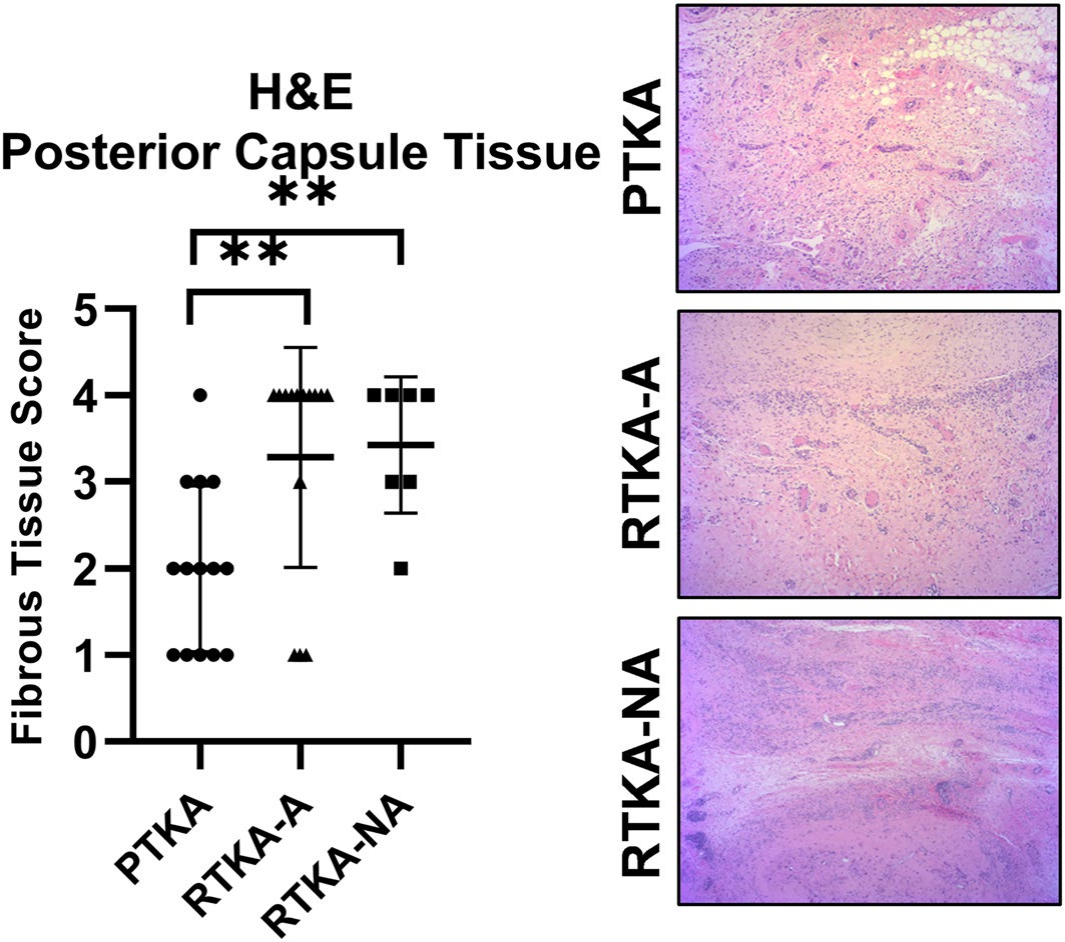
Histopathologic assessment of fibrosis in the periarticular knee tissue. Histopathologic fibrotic scores of H&E-stained sections for the periarticular tissue and representative images from the three patient groups (*PTKA* = primary TKA, *RTKA-A* = arthrofibrosis, *RTKA-NA* = non-arthrofibrosis). The graphs depict mean fibrous tissue score ± standard deviation, with each data point representing one patient. Asterisk denotes a significant difference, (**p ≤ 0.01), by the Wilcoxon Rank Sum test. All images were taken using the 10X objective on a Nikon E600 bright-field light microscope.

Blinded histopathologic assessment of fibrosis in the periarticular tissue on H&E-stained sections showed significant histological differences among the three groups (Fig. 2). The mean histopathologic fibrosis score for primary TKA patients was 2.0 and the mode score was 2, arthrofibrotic revision patients was 3.3 and the mode score was 4, and non-arthrofibrotic revision patients was 3.4 and the mode score was 4. Accordingly, increased amounts of fibrous tissue were evident for both the fibrotic revision and non-fibrotic revision groups of patients when compared to the primary TKA group (Kruskal-Wallis p = 0.005 for both). The fibrosis scoring did not reveal any association between histopathologic score and patient age, sex, BMI or KSS.

### Analysis of collagen content using picrosirius red

**Figure 3:**
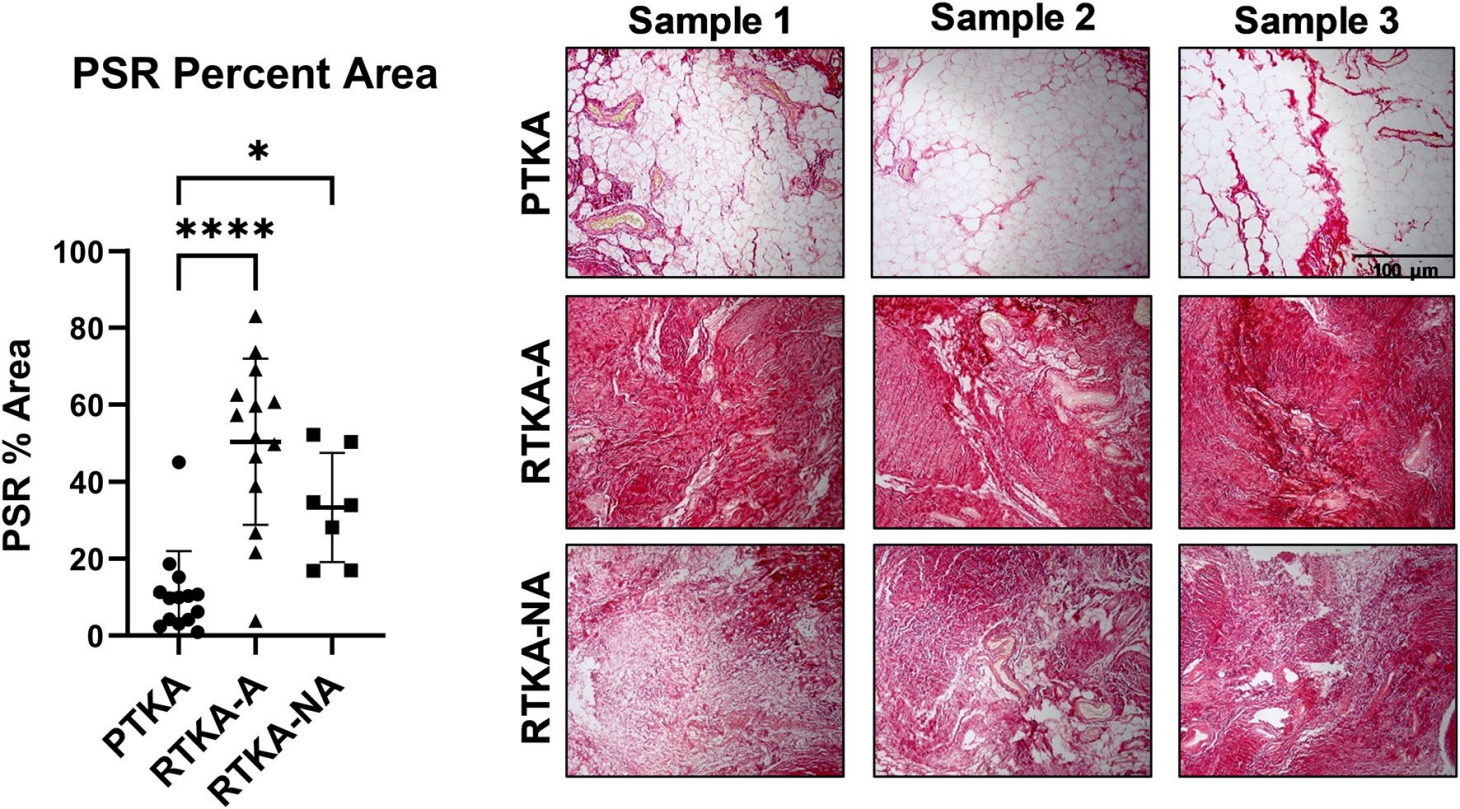
Picrosirius red staining of periarticular knee tissue. Picrosirius red staining quantification and representative images of three patients from the three patient groups (*PTKA* = primary TKA, *RTKA-A* = arthrofibrosis, *RTKA-NA* = non-arthrofibrosis). The graphs depict mean picrosirius red staining (PSR) percent area ± standard deviation, with each data point representing one patient (average of three areas). Asterisk denotes a significant difference, (*p ≤ 0.05; ****p ≤ 0.0001), by the Wilcoxon Rank Sum test. The scale bar applies to all images in this figure.

Evaluation of collagen deposition was assessed by Picrosirius red staining (Fig. 3, Supplementary Fig. 1). The percent positive staining area for the arthrofibrosis revision group was 50.4% compared to 10.9% in the primary TKA group (p < 0.0001) and 33.4% in the revision non-arthrofibrotic group (p = 0.047). No correlations were found between any of the picrosirius red staining data and patient age, BMI, sex or KSS. Thus, collagen content differs among posterior capsule specimens primarily based on surgical group.

### Immunofluorescence microscopy analysis for ACTA2 + /Lamin- myofibroblast analysis

**Figure 4:**
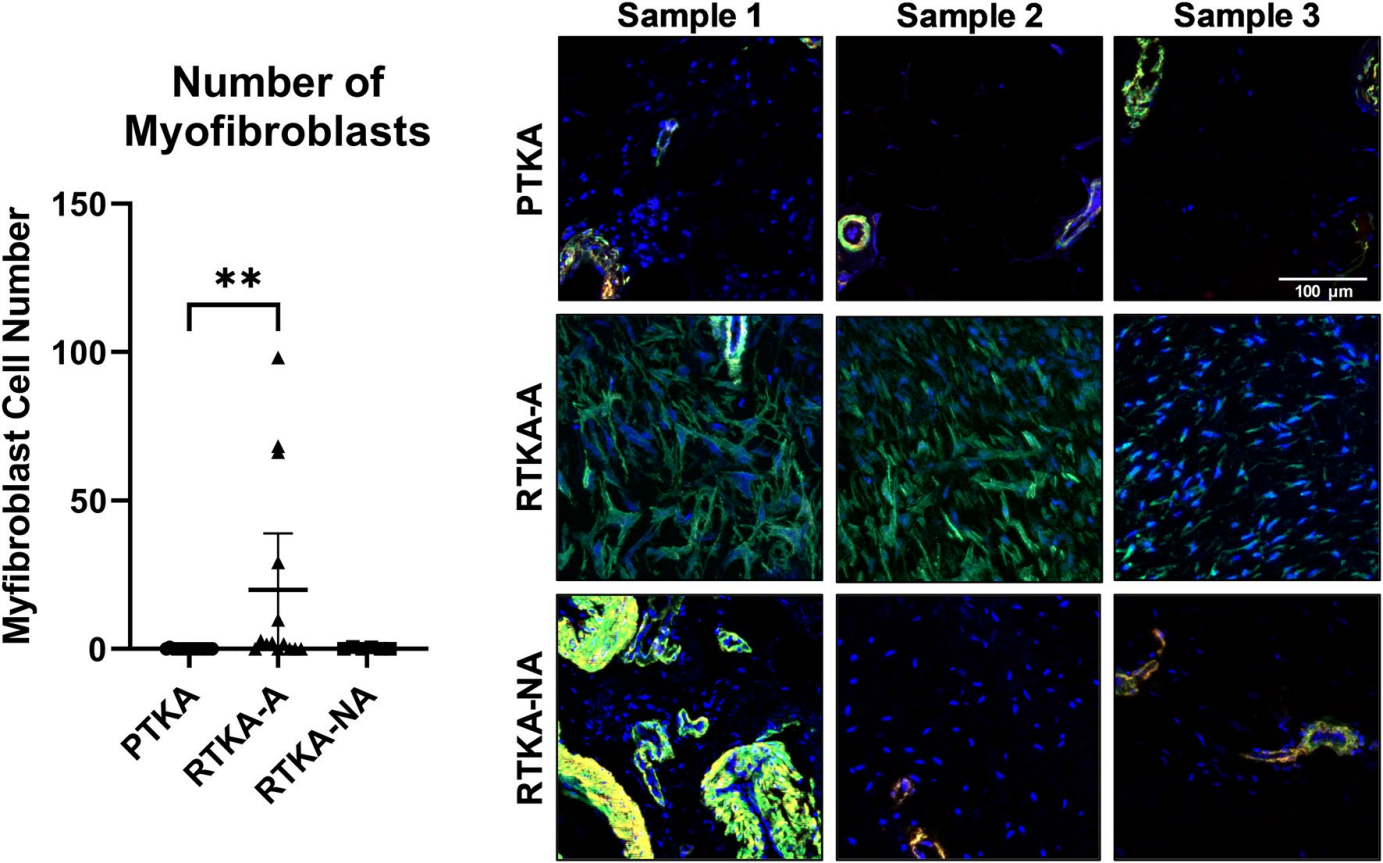
Myofibroblast quantification of periarticular knee tissue. Myofibroblast quantification and representative immunofluorescence merged images for α-SMA (green), Laminin (red) and DAPI (blue) from the three patient groups (*PTKA* = primary TKA, *RTKA-A* = arthrofibrosis, *RTKA-NA* = non-arthrofibrosis). Sample 1, Sample 2, and Sample 3 depict images from three different patients for each group. The graphs depict mean myofibroblast number defined as ACTA2 + /Laminin- per area ± standard deviation, with each data point representing one patient. Two asterisks denote a significant difference, p ≤ 0.01, by the Wilcoxon Rank Sum test. The scale bar applies to all images in this figure.

Immunofluorescence microscopy for ACTA2 (myofibroblast marker) and Laminin (blood vessel marker) revealed a significant increase in ACTA2 + /Lamin- cells in the arthrofibrosis revision group (mean = 20 ± 33 cells/area) compared to the primary TKA group (mean = 0.04 ± 0.1 cells/area; p = 0.002; *d* = 0.86, Fig. 4, Supplementary Fig. 2). The non-arthrofibrotic revision group also had a lower mean number of ACTA2 + /Lamin- cells (mean = 0.2 ± 0.3 cells/area; p = 0.06; *d* = 0.73) when compared to the arthrofibrosis revision group, although this difference did not reach statistical significance. The ICCs revealed good interobserver reliability (interclass correlation = 0.80, p < 0.001) and excellent intraobserver reliability (intraclass correlation = 0.97, p < 0.001). No association was found between the number of myofibroblasts (i.e., ACTA2 + /Lamin- cells) and patient age, BMI, sex or KSS. Hence, posterior capsule specimens exhibit arthrofibrosis related distinctions in myofibroblast content.

### Microscopic analysis of immune cell content

To assess for alterations in immune cell populations, macrophages (CD163 +), mast cells (CD117 +), T-cells (CD3 +), and B-cells (CD20 +) were counted in primary TKA, arthrofibrosis revision, and non-arthrofibrosis revision tissues (Table 3). Because whole tissues sections are heterogenous and specific tissue regions may be prone to immune cell alterations, we also assessed specific changes in the immune cell profiles within the fibrous, adipose and synovium regions of tissue sections.

**Table 3:** Mean cell densities for CD163 + macrophages, CD117 + mast cells, CD3 + T-cells and CD20 + B-cells in the whole tissue and in the fibrous, adipose and synovial tissue regions.

Marker	Region	PTKA mean cell density (SD)	RTKA-A mean cell density (SD)	RTKA-NA mean cell density (SD)	Kruskal–Wallis p-value	p-value PTKA vs. RTKA-A	p-value RTKA-NA vs. RTKA-A	p-value PTKA vs. RTKA-NA
CD163	Overall	413.0 (298.9)	251.7 (131.6)	673.7 (938.0)	0.585	0.246	0.856	0.971
	Fibrous	474.3 (249.5)	240.9 (140.8)	598.3 (512.5)	**0.042**	**0.011**	0.179	0.841
	Adipose	222.9 (149.6)	278.9 (142.6)	104.4 (47.0)	0.103	0.287	**0.033**	0.197
	Synovium	3004.6 (1225.3)	1796.4 (1273.1)	3623.0 (0)	**0.030**	0.069	N/A*	N/A*
CD117	Overall	13.2 (10.2)	18.4 (19.8)	10.9 (9.9)	0.760	0.804	0.535	0.585
	Fibrous	27.2 (31.4)	21.7 (21.0)	15.0 (12.0)	0.838	0.982	0.718	0.547
	Adipose	6.3 (4.7)	12.3 (8.5)	10.4 (11.9)	0.282	0.094	0.999	0.781
	Synovium	59.9 (66.9)	3.6 (4.1)	14.6 (0)	**0.003**	**0.003**	N/A*	N/A*
CD3	Overall	2.7 (3.0)	4.8 (5.5)	9.3 (9.9)	0.092	0.104	0.353	0.078
	Fibrous	4.7 (6.1)	3.7 (4.4)	9.1 (9.8)	0.283	0.650	0.179	0.179
	Adipose	1.3 (1.7)	6.5 (7.0)	1.4 (2.0)	0.195	0.084	0.283	0.916
	Synovium	9.8 (12.9)	0.4 (0.6)	12.4 (0)	0.170	0.167	N/A*	N/A*
CD20	Overall	6.8 (8.6)	3.3 (5.0)	7.9 (13.2)	0.326	0.141	0.701	0.547
	Fibrous	9.9 (11.3)	4.0 (7.5)	8.2 (13.1)	0.542	0.350	0.416	0.904
	Adipose	1.4 (1.2)	3.5 (4.8)	1.0 (1.8)	0.527	0.487	0.450	0.429
	Synovium	32.3 (58.1)	0.95 (1.3)	6.6 (0)	0.224	0.156	N/A*	N/A*

Significant values are in bold.

*PTKA* = primary TKA, *RTKA-A* = revision TKA for arthrofibrosis, *RTKA-NA* = revision TKA for non-arthrofibrotic reasons *SD* = standard deviation.

*There was only one RTKA-NA sample with synovial tissue; unable to perform statistical analysis with only one sample.

**Figure 5:**
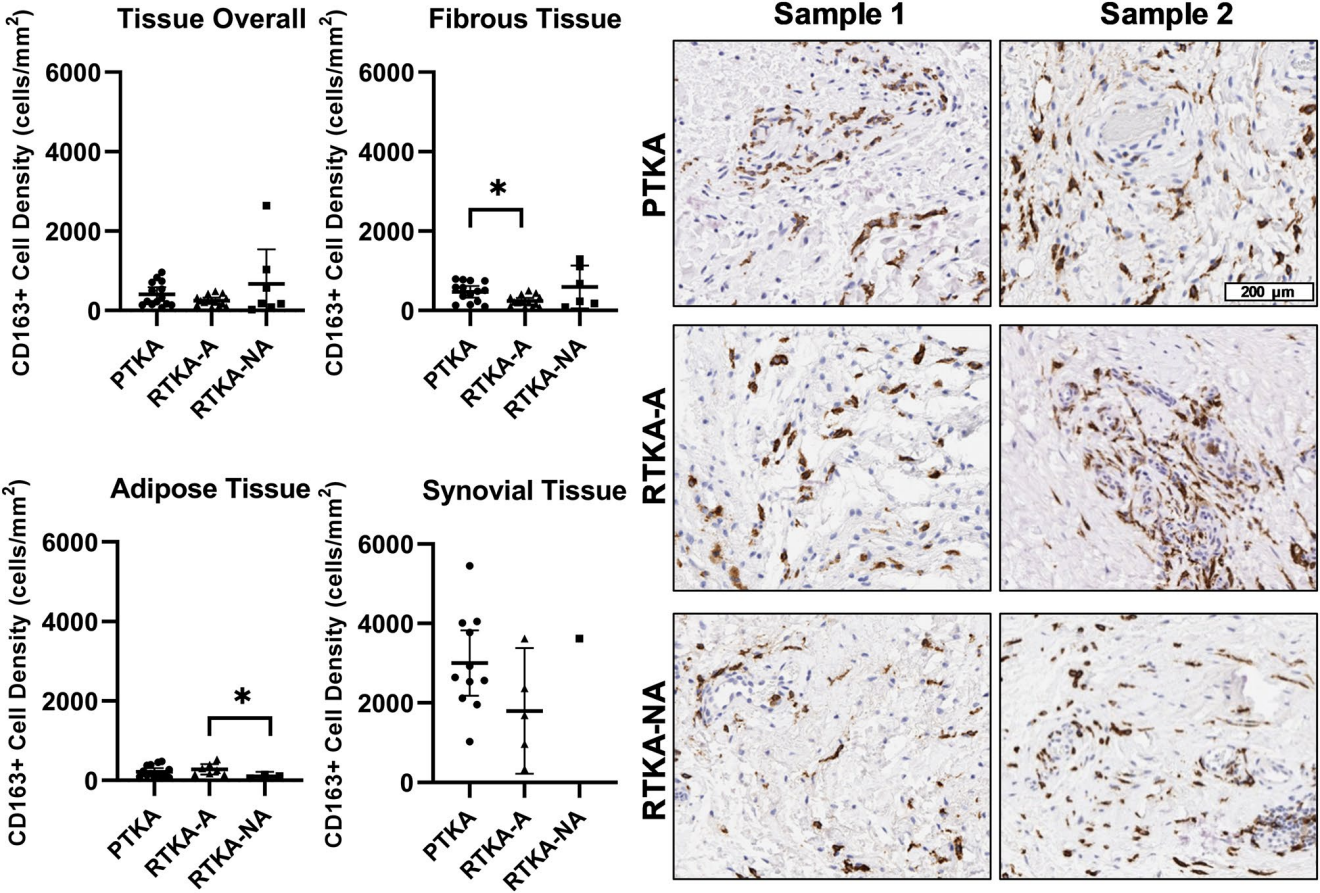
Quantification of macrophages in periarticular knee tissue. Tissue density of CD163 + macrophages per mm^2^ in the overall tissue as well as the fibrotic, adipose, and synovium regions with representative images from the three groups (*PTKA* = primary TKA, *RTKA-A* = arthrofibrosis, *RTKA-NA* = non-arthrofibrosis). The graphs depict mean macrophage density (CD163 + cell/mm^2^) ± standard deviation, with each data point representing one patient. Asterisk denotes a significant difference, p ≤ 0.05, by the Wilcoxon Rank Sum test. Sample 1 and Sample 2 depict images from two different patients in the group. The scale bar applies to all images in this figure.

In whole tissue sections, CD163 + macrophages were the most abundant immune cells in posterior capsule tissues from all three patient groups, with the synovial region containing the highest CD163 + macrophage densities of all tissue regions (Table 3). CD163 + macrophage densities did not significantly differ in whole tissue sections across the three groups: the mean cell density (cells/mm^2^ of tissue) was 412 ± 299 for primary TKA group, 252 ± 132 for arthrofibrotic revision group, and 674 ± 938 for the non-arthrofibrotic revision group (Kruskal-Wallis p-value = 0.6; Table 3, Fig. 5). Across different tissue regions (fibrous, adipose, and synovium), there were significant differences in CD163 + cell density/mm^2^ (Kruskal-Wallis p-values = 0.04, 0.1, and 0.03, respectively; Fig. 5). In the fibrous tissue, the arthrofibrotic revision patients had a significantly lower CD163 + macrophage density/mm^2^ (mean = 241 ± 141) compared to the primary TKA (mean = 474 ± 250; p = 0.01; *d* = 1.15). A similar trend was observed when looking at CD163 + staining density in the synovium comparing the primary TKA (mean = 3005 ± 1225) and arthrofibrotic revision groups (mean = 1796 ± 1273; p = 0.07; *d* = 0.97), although this was not statistically significant. There were no significant correlations between CD163 + macrophage density and patient age in both fibrous (r = 0.34, p = 0.05) and adipose tissues (r = 0.39, p = 0.05) across all three patient groups. However, among non-arthrofibrotic revision patients, age had a striking strong correlation with CD163 + cell density (r = 0.99, p = 0.006). Likewise, CD163 + macrophage cell density and BMI were positively correlated in the synovial tissue of the arthrofibrotic revision groups, although this was not statistically significant (r = 0.90, p = 0.08). Across all three groups, an increase in CD163 + macrophage density was associated with a higher preoperative KSS (r = 0.35, p = 0.04). In sum, CD163 + macrophages were the most abundant immune cell type in any capsular sample, with a significant decrease in the fibrous tissue region of the arthrofibrotic revision specimens.

**Figure 6:**
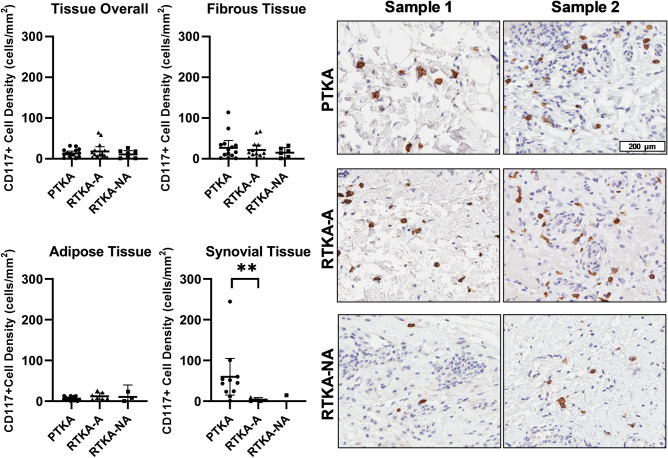
Quantification of mast cells in periarticular knee tissue. Tissue density of CD117 + mast cells per mm^2^ in the overall tissue and in the fibrotic, adipose and synovium regions with representative images from the three groups (PTKA = primary TKA, RTKA-A = arthrofibrosis, RTKA-NA = non-arthrofibrosis). The graphs depict mean mast cell density (CD117 + cell/mm^2^) ± standard deviation, with each data point representing one patient. Two asterisks denote a significant difference, p ≤ 0.01, by the Wilcoxon Rank Sum test. Sample 1 and Sample 2 depict images from two different patients in the group. The scale bar applies to all images in this figure.

Mast cells, which are characterized as CD117 + cells, showed a much more varied expression pattern when compared to CD163 + macrophages and myofibroblasts. The overall (all tissue regions combined) CD117 + mast cell numbers (cells/mm^2^ tissue) were similar among all three groups: mean 13 ± 10 for the primary TKA, 18 ± 20 for arthrofibrotic revision, and 11 ± 10 for non-arthrofibrotic revision groups (Kruskal-Wallis p-value = 0.8; Table 3, Fig. 6). Similarly, no appreciative differences were noted in mast cell numbers between primary TKA, non-arthrofibrotic revision, and arthrofibrotic revision in fibrous and adipose tissue areas. However, there was a much lower density of CD117 + cells in the synovium of the arthrofibrotic revision group (mean = 4 ± 4) compared to the primary TKA group (mean = 60 ± 67; p = 0.07; *d* = 1.19), although this difference did not achieve statistical significance. Of note, there was insufficient synovium in the tissue samples from the non-arthrofibrotic revision cohort to compare this region with the other two groups. The CD117 + mast cell density in the fibrotic tissue region showed a moderate correlation with BMI (r = 0.57, p = 0.03), but only in the arthrofibrotic revision group. There was no association between CD117 + mast cell densities and patient age, sex or KSS. Taken together, these data established mast cell density is reduced within the synovial tissues of arthrofibrotic revision group when compared to primary TKA group.

**Figure 7:**
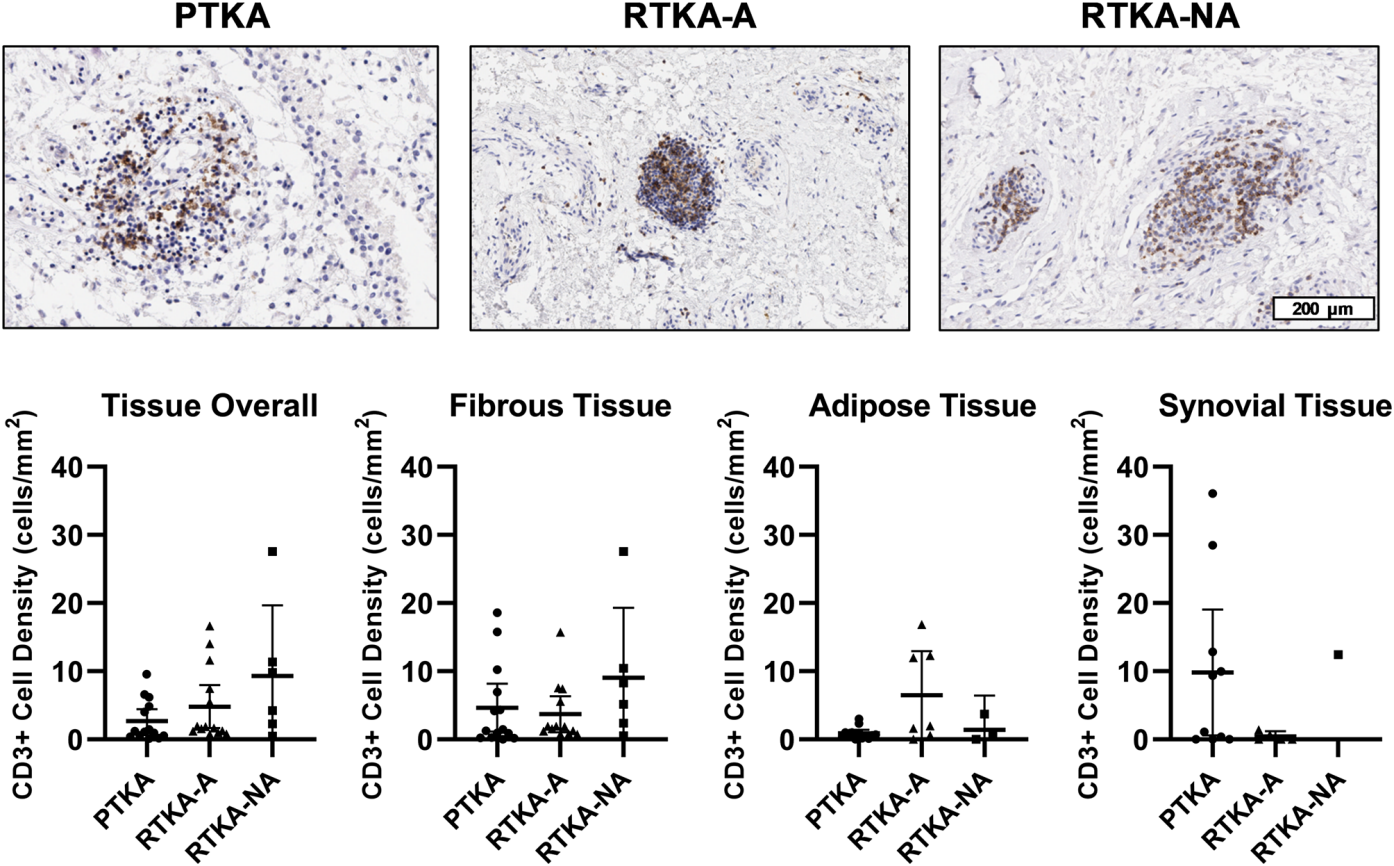
Quantification of T-cells cells in periarticular knee tissue. Tissue density of CD3 + T-cells per mm^2^ in the overall tissue and in the fibrotic, adipose and synovium regions with representative images from the three groups (*PTKA* = primary TKA, *RTKA-A* = arthrofibrosis, *RTKA-NA* = non-arthrofibrosis). The graphs depict mean T-cell density (CD3 + cell/mm^2^) ± standard deviation, with each data point representing one patient. The scale bar applies to all images in this figure.

In addition to macrophages and mast cells, we also counted the number of T cells (CD3 +) and B cells (CD20 +) cells in the three groups of tissues. Overall, the T cell density (CD3 + cells/mm^2^ tissue) in the primary TKA group (3 ± 3) was lower than arthrofibrotic (5 ± 6) and non-arthrofibrotic (9 ± 10) revision groups; however, these differences did not reach statistical significance (Kruskal-Wallis p-value = 0.09). While some changes in the number of T cells within adipose and synovial tissues regions between primary TKA and arthrofibrosis revision samples was observed, neither of these tissue areas reached statistical significance (p-values = 0.084 and 0.167, respectively) (Fig. 7, Table 3). Generally, females had higher CD3 + cell density when compared to males in the fibrotic tissue compartment (p = 0.08). However, this trend was not significant and was not consistently observed in the overall tissue or the other compartments. No correlations were noted between the number of CD3 + T-cells and patient age, BMI or KSS.

**Figure 8:**
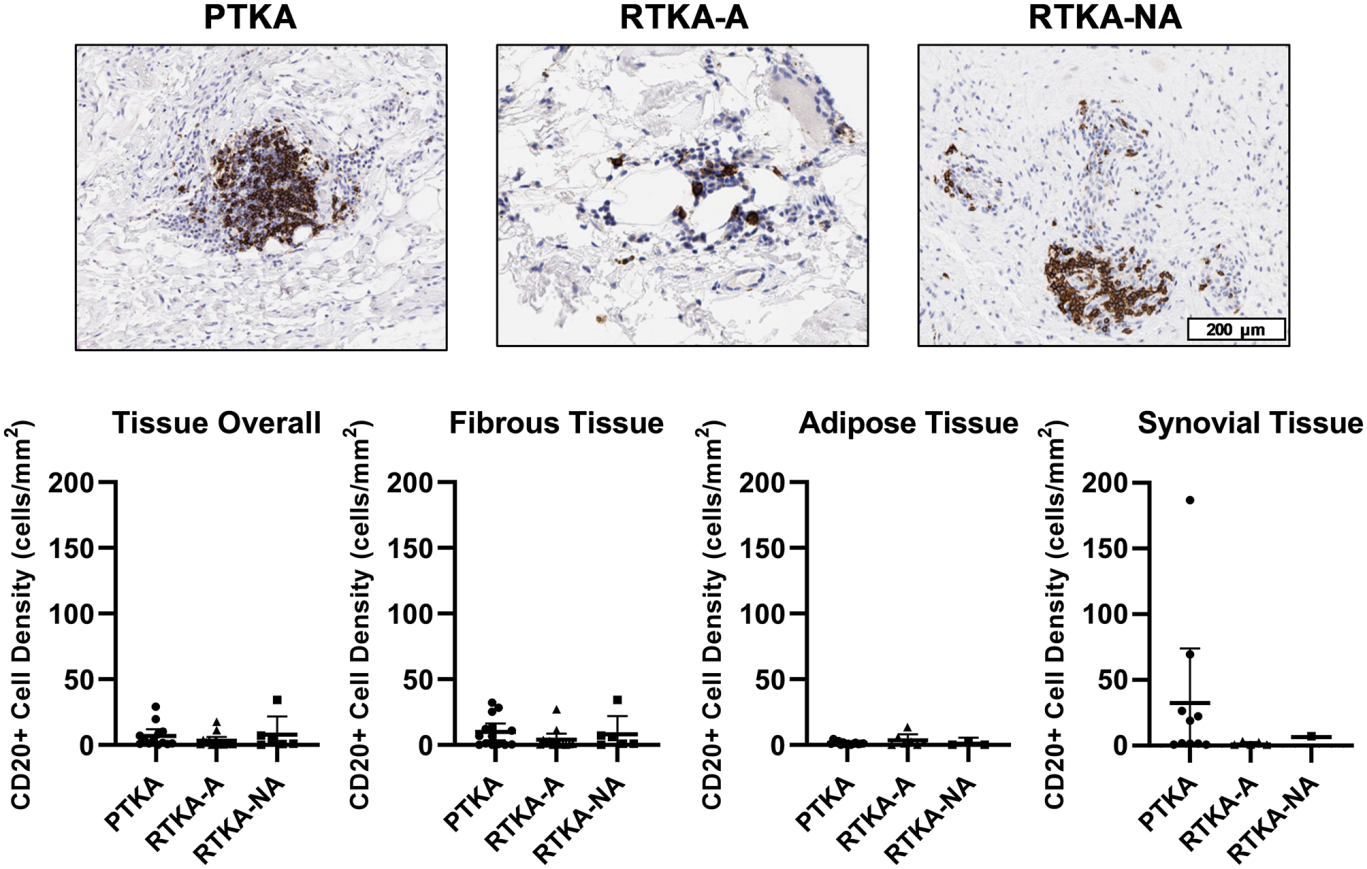
Quantification of B-cells cells in periarticular knee tissue. Tissue density of CD20 + B-cells per mm^2^ in the whole tissue and in the fibrotic, adipose and synovium regions with representative images from the three groups (PTKA = primary TKA, RTKA-A = arthrofibrosis, RTKA-NA = non-arthrofibrosis). The graphs depict mean B-cells density (CD20 + cell/mm2) ± standard deviation, with each data point representing one patient. The scale bar applies to all images in this figure.

As with T cells, CD20 + B cell densities (CD20 + cells/mm^2^ tissue) were similar across all three groups within the overall tissue samples (7 ± 9 for primary TKA group, 3 ± 5 for the arthrofibrotic revision group, and 8 ± 13 for the non-arthrofibrotic revision group, (Kruskal-Wallis p-value = 0.33) (Fig. 8, Table 3). The highest density of CD20 + B-cells was seen in the synovial tissue region for the primary TKA group, albeit with high standard deviation. Across all groups, there was a negative correlation between CD20 + B cell density and patient age in the adipose tissue region (r = − 0.48, p = 0.02). In contrast, BMI was positively correlated with CD20 + B cell density in the adipose tissue region across all groups (r = 0.39, p = 0.05). Preoperative KSS for patients in the non-arthrofibrotic revision group was highly correlated with the CD20 + B cell density in the whole tissue samples and in the fibrous tissue region (both r = 0.94, p = 0.02). Together, these data revealed that in our three study groups, there was no significant differences with CD3 + T-cells and CD20 + B-cells.

### Correlation between immune cell numbers and gene expression

**Figure 9:**
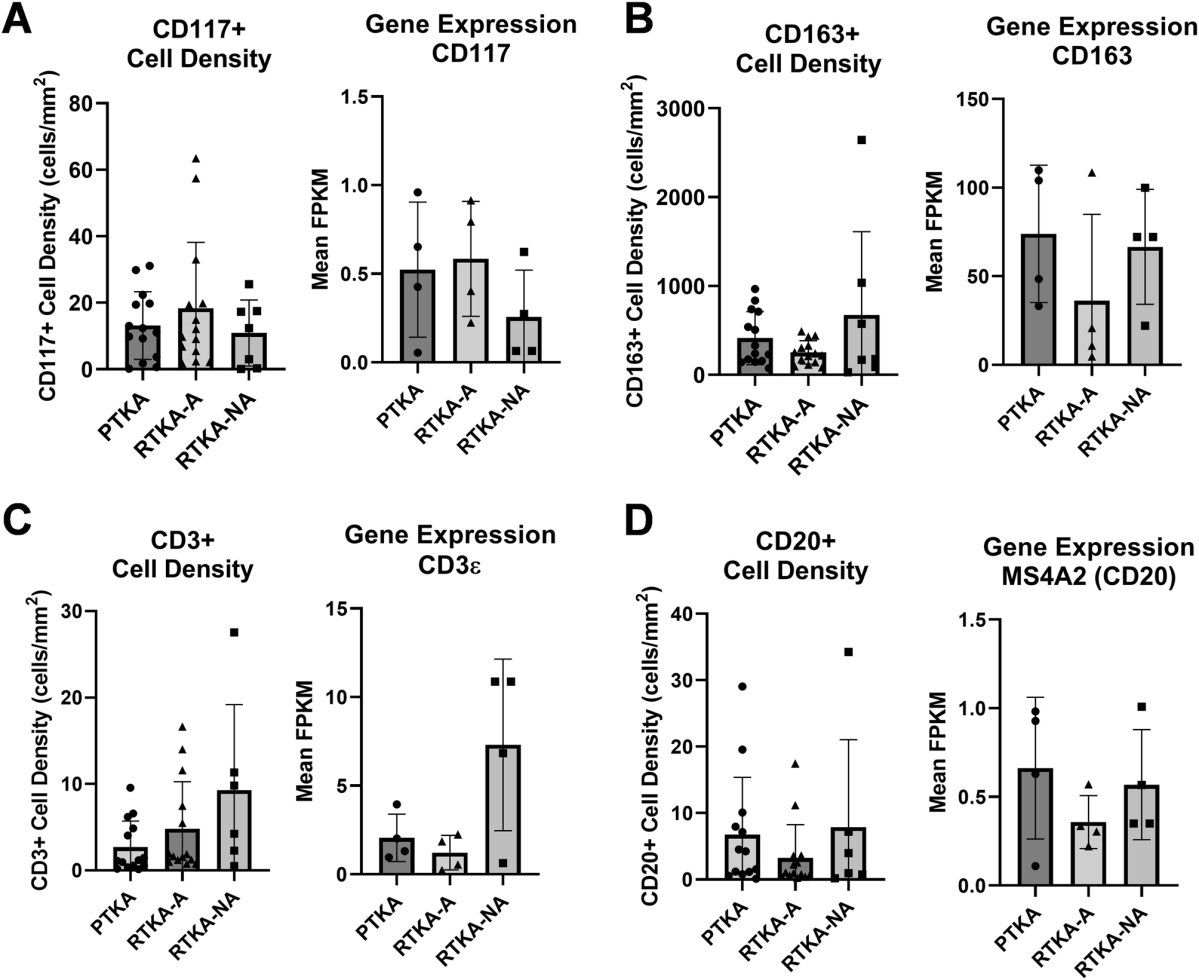
Correlation between immune cell numbers and gene expression in periarticular knee tissue. Tissue density of CD117 + mast cells (**A**), CD163 + macrophages (**B**), CD3 + T-cells (**C**), and CD20 + B-cells (**D**) in whole tissues in relationship to mRNA expression levels of the corresponding genes as assessed by RNA-Seq analysis from bulk tissue. The graphs depict mean cells densities (# of cells/mm2) ± standard deviation and mean gene expression (FPKM) ± standard deviation, with each data point representing one patient.

Examining the mean FPKMs expression for CD117 (KIT), CD163, CD3_ε_, and MS4A2 (CD20), from our previous RNA-sequencing data^24^, we observed similar trends between the gene expression (RNA-seq) and the overall cell density for CD117 +, CD163 +, CD3 + and CD20 +. This was observed for all three patient groups, including primary TKA, arthrofibrotic revision TKA, and non-arthrofibrotic revision TKA (Fig. 9). Taken together, the trends observed in the immune cell numbers defined by specific CD markers is correlative with RNA expression levels of these particular cell markers in the periarticular knee tissue samples.

## Discussion

The present study demonstrated significant variability in tissue fibrosis and the immune cell microenvironments among patients undergoing primary TKAs, those needing revision TKAs for arthrofibrosis, and those undergoing revision TKAs for non-fibrotic and non-infectious etiologies. CD163 + expressing macrophages were the most abundant immune cell type in all specimens for all cohorts, yet the overall density was lower in the arthrofibrotic revision knees compared to the primary TKA. These immune cells were also found in different densities in different regions between these groups with particularly high densities within the synovial regions of the primary TKA, but lower densities in the fibrotic regions of the arthrofibrotic revision knees.

Routine histopathologic scoring of the H&E-stained tissue sections revealed no significant difference between the arthrofibrosis revision group and the primary TKA and non-arthrofibrotic revision control groups. Yet, a significant difference was observed between the degrees of fibrosis in the primary TKA group compared to the two revision TKA groups. This result may have emerged because the revision surgeries (either for arthrofibrotic or non arthrofibrotic reasons) represent a second surgical procedure for these patients, and scar/fibrotic tissue has already developed as a result of the index TKA.

Conversely, a significant increase in collagen deposition was noted with the arthrofibrotic revision group when compared to the two control groups. These data correspond with the clinical finding that arthrofibrotic knees develop quantitatively more scar tissue and a different quality of scar tissue than a primary TKA or a revision non-arthrofibrotic TKA^27^. The non-arthrofibrotic revision specimens likely do have some scar tissue present from their previous surgery, however there is significantly more of this scar tissue in the arthrofibrotic revision specimens. The difference between the arthrofibrotic and non-arthrofibrotic groups was likely underappreciated by H&E analysis due to the evaluation being based on a scoring system.

Likewise, a significantly higher number of myofibroblasts were observed in the arthrofibrotic revision tissues when compared to the primary TKA tissues. Our group has previously published on the role of myofibroblasts, especially in the first few weeks, in a rabbit contracture model of arthrofibrosis^10,28^. Abdul and colleagues^9^ reported a similar finding of significantly increased α-SMA expression in their human synovial membrane and infrapatellar fad pad specimens of arthrofibrotic and non-arthrofibrotic revision patients compared to the primary TKA patients. However, no significant differences were detected between the arthrofibrotic revision group and the non-arthrofibrotic revision group, nor between the primary TKA group and the non-arthrofibrotic revision group. This observation may be due to the limited size of non-arthrofibrotic revision TKA group (n = 7) in this study and/or the varying intervals between the index surgery and revision surgery. The mean time between primary and revision TKA in the arthrofibrosis group was 3 years, while the mean time between primary and revision TKA in the non-arthrofibrotic group was 7 years. Currently, there is no standardized treatment interval from primary TKA to revision TKA when treating an arthrofibrotic patient. Several factors can affect this duration, including the use of other treatment strategies such as physical therapy and/or MUA prior to revision surgery.

Our current data do suggest that quantitative digital image analysis of selected immune cell populations in biopsy specimens may prove helpful in selecting surgical patients if threshold values can be established. Specifically, CD117 + mast cells were decreased in the synovium of the arthrofibrotic revision group and found in greater density within fibrotic areas compared to the primary TKA group. The role of mast cells, which express various fibroblast growth factors^29–31^, have been studied in both pulmonary and cardiac fibrosis^11,12^. It has also been reported that tissues from arthrofibrotic patients have increased numbers of mast cells^32^, and in a rabbit model of posttraumatic joint contractures, mast cell stabilizers (e.g., ketotifen) have been shown to help improve motion^20,33^. We propose that the decrease in CD117 + cells observed in the synovium of arthrofibrotic revision patients in this study is due to samples being taken at much later time points in the pathogenesis of patients in our series undergoing revision TKA for arthrofibrosis as opposed to the subjects in our prior animal studies^10,28,34–39^. Mast cells are some of the first cell types to respond to injury or surgical insult^31^. They can have a late-phase reaction up to 24 h after the immediate reaction, but persistence beyond this is uncommon^40^. As such, at the time these tissue samples were taken, which for the arthrofibrosis revision group was up to 10 years after the primary TKA, mast cells had likely completed their innate immune response to the initial injury. Additionally, mast cells have been shown to be increased in obese patients^41^. This finding is consistent with a positive correlation we observed between CD117 + cell density and BMI in arthrofibrosis revision patients in the present study.

Macrophages have been studied in other forms of fibrosis due to their production of potent pro-fibrotic cytokines^12^. To our knowledge, they have not been studied in arthrofibrosis specifically, although these immune cells may be fundamental to the pathophysiology of rheumatoid arthritis^42^. Our data demonstrate a trend of decreased CD163 + macrophages in the arthrofibrotic revision group compared to the primary TKA and non-arthrofibrotic revision groups. Macrophages have been described to play a role in wound healing and tissue repair^15,43,44^. The correlation identified in this study between increased patient age and increased CD163 + cell density may be associated with the low-grade inflammation that occurs in many organs including periarticular tissues with aging. Some studies suggest that there is a shift in macrophage polarization from M2 functions (tissue repair and wound healing) to an M1 signature (inflammation and tissue destruction) during aging^45,46^. Few studies have recognized the importance of macrophage polarization on fibrosis^47,48^. We identified a positive correlation between CD163 + macrophage tissue density and the preoperative patient-reported outcome of KSS. Similar to mast cells, BMI was found to have a positive correlation with CD163 + cell density in the synovial tissue region. This correlation is likely due to the adipose-related inflammation, which has been reported to affect macrophage populations^49^. Like the mast cells however, their role likely occurs at an earlier time point during the evolution of arthrofibrosis which may explain why higher densities are not observed in the chronic arthrofibrotic patient.

The role of T-cells in arthrofibrosis has been previously described^50^, although this prior study examined patients undergoing arthrolysis of the knee for arthrofibrosis after ligament injuries rather than revision TKA. The mean time between trauma and arthrolysis surgery was 16 months, which is much less than our current study. Using several IHC markers, their study showed increased T-cell numbers in the arthrofibrosis patients compared to the control tissues, taken from knees with normal macroscopic evaluation of the synovium. Our study did not corroborate these results as we did not observe significant differences between the revision TKA groups and the primary TKA group. However, we did notice an increase of CD3 + T-cells in female patients versus male patients. It has been suggested that hormones, specifically estrogen levels, play a role in regulation and modulation of immune responses^51^. Hence, hormone levels may account for differences in detection of T-cells between distinct studies^51^.

Similar to T-cells, our data did not reveal a difference in B-cell density between the patient groups. We observed a parallel trend to CD3 + T cell density in that female patients in our study had a greater CD20 + B-cell density than males. We also observed a negative correlation between age and CD20 + B cells, which is consistent with the literature on immunosenescence^52,53^. Furthermore, comparable with mast cells and macrophages in this study, increased CD20 + cell density was associated with increased BMI, likely due to mast cells being present in adipose tissue and the increased number of these cells in obese patients^41^. We also identified a positive correlation between preoperative KSS and CD20 + cell density in the non-arthrofibrosis revision group. We postulate that this difference in CD20 + cells may be related to reduced pain and knee function preoperatively in patients with higher KSS and better innate immune responses that accompany their various clinical complications.

The transcriptome (RNA-Seq) of periarticular tissues of patients undergoing primary TKAs, those needing revision TKAs for arthrofibrosis, and those undergoing revision TKAs for non-fibrotic and non-infectious etiologies has been previously reported by our group^24^. In the present study, we correlated immune cell numbers as defined by CD marker presentation on the cell surface from the present study to the overall expression patterns of mRNAs for these particular markers performed in the earlier study^24^. Of note, all of the tissue samples that were assessed for gene expression (four samples for each cohort)^24^ are included in the present study. However, additional patient samples have been included in the present study to assess the immune cell content of periarticular tissues. Of interest, our data analysis revealed similar trends when comparing the number of immune cells as defined by positive staining for select CD markers and the RNA levels of genes that encode these CD markers by RNA-seq analysis. Thus, gene expression patterns of CD markers within periarticular tissues may be employed in the future as a proxy to the counting of immune cells based on the presentation of these CD markers on the cell surface. In support, previous studies by our group have established a correlation between the mRNA levels and presentation of CD markers on the cell surface of adipose-derived mesenchymal stem cells^54^.

This histopathological study provides new insights into the histopathology of arthrofibrosis but our findings have limitations. The small sample size in each of the three groups, with the smallest group containing 7 specimens (non-arthrofibrotic revision control group), may have underpowered the various analyses. The non-arthrofibrotic revision group was the most difficult to match to the arthrofibrotic samples due to the strict inclusion criteria of no history of infection or fibrosis, as well as matching age, sex, BMI, smoking and diabetes status. The influence of the small sample size can also be observed in the effect size calculations (Supplementary Table 2). In addition, this study only examined the tissue reaction taking place in the posterior capsule tissue of the knee. Future studies should incorporate histopathological examination of other regions of the knee joint. For example, it has been proposed that the synovium of the suprapatellar pouch or the epitenon of the quadriceps tendon may play a role in the development of fibrosis^55,56^. The posterior capsule specimens were taken by various surgeons at our institution, thus subtle differences in sampling site among surgeons might have contributed to cellular variability in tissue composition. When analyzing the tissue by region, this sampling variation was evident based on different ratios of connective tissue to adipose to synovium in each of the three patient groups. Finally, this study only utilized a few cell type-specific IHC stains and conventional microscopic evaluation (manual or digital image analysis) to begin characterizing the cellular features of arthrofibrosis. Further molecular analyses, both by microscopy and by measurements in tissue homogenates, will be needed to explore disease-related mechanisms that contribute to arthrofibrosis.

In summary, this study describes the tissue cell densities of α-SMA + /Lamin- myofibroblasts, CD117 + mast cells, CD163 + macrophages, CD3 + T-cells and CD20 + B-cells in different tissue regions of the posterior capsule of arthrofibrosis revision patients and compares these cell densities to primary TKA and non-arthrofibrotic revision TKA specimens. Based on our data, specific immune and stromal cell populations in posterior capsule tissues may prove useful as biological markers for the characterization of arthrofibrosis, including CD117/KIT + mast cells, CD163 + macrophages and the ACTA2 + (α-SMA)/Lamin- myofibroblasts. This study provides an important snapshot of the cell types involved in arthrofibrosis, which provides insight into the pathogenesis and development of this disease and may potentially influence treatment strategies. Assessment and quantification of these cell types in biopsies might aid in determining future treatment algorithms for patients requiring surgical intervention.

## Supplementary Information

Supplementary Figures.

Supplementary Table 1.

Supplementary Table 2.

## Data Availability

The data that support the findings of this study are available in Gene Expression Omnibus (GEO) database at https://www.ncbi.nlm.nih.gov/geo/, Accessions #GSE135854.
