# Supplementary material for: Immune cell populations differ in patients undergoing revision total knee arthroplasty for arthrofibrosis
Source: Sci Rep. 2022 Dec 31;12:22627. doi: 10.1038/s41598-022-22175-3 (PMC9805429; doi:10.1038/s41598-022-22175-3)
Supplement: Supplementary file 1 — Supplementary Figures. [file 41598_2022_22175_MOESM1_ESM.pptx]

## Slide 1
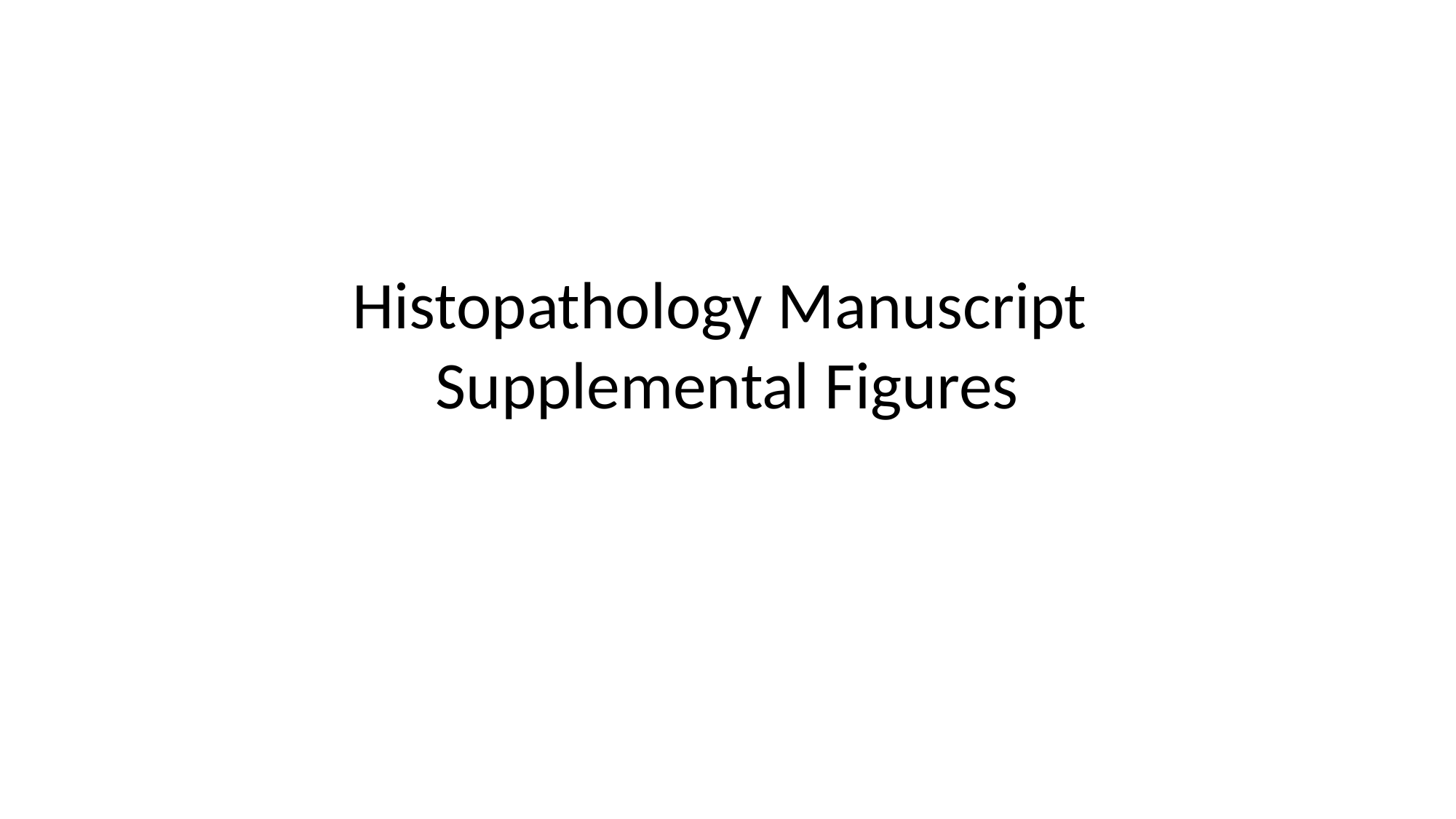

# Histopathology Manuscript Supplemental Figures

## Slide 2
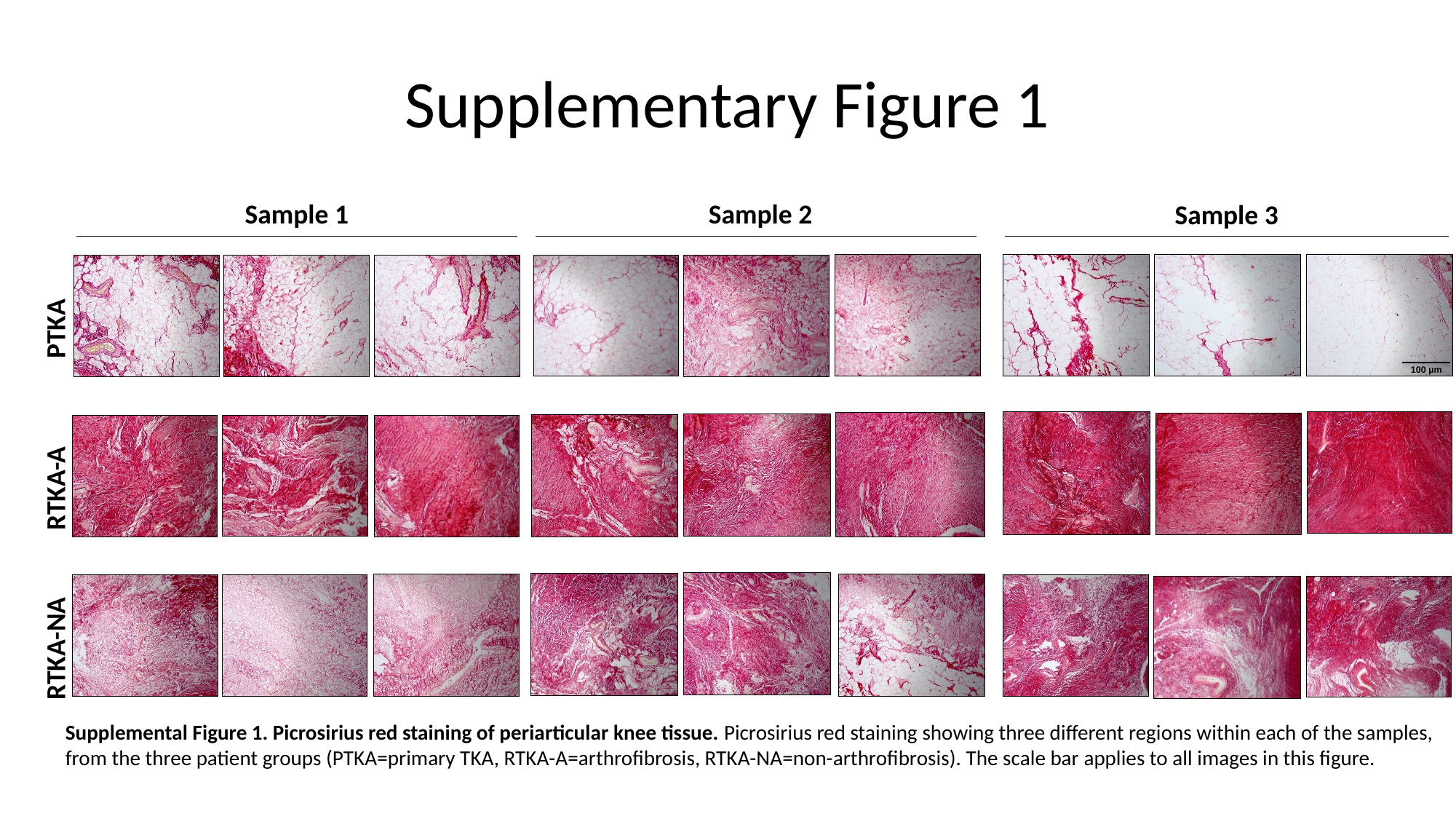

# Supplementary Figure 1
Sample 1
Sample 2
Sample 3
PTKA
100 µm
RTKA-A
RTKA-NA
Supplemental Figure 1. Picrosirius red staining of periarticular knee tissue. Picrosirius red staining showing three different regions within each of the samples, from the three patient groups (PTKA=primary TKA, RTKA-A=arthrofibrosis, RTKA-NA=non-arthrofibrosis). The scale bar applies to all images in this figure.

## Slide 3
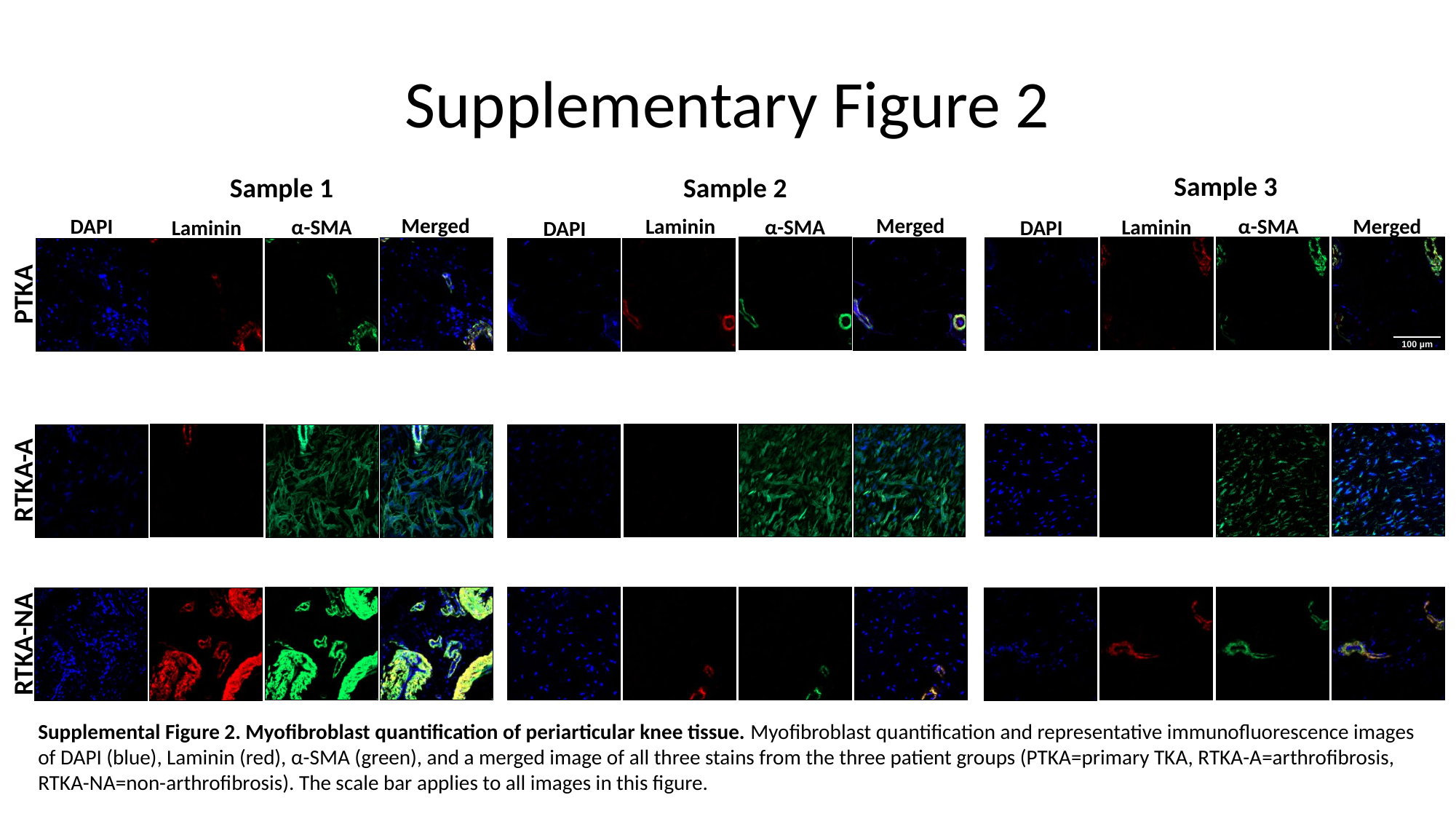

# Supplementary Figure 2
Sample 3
Sample 1
Sample 2
Merged
Merged
DAPI
α-SMA
Laminin
Merged
α-SMA
Laminin
α-SMA
Laminin
DAPI
DAPI
PTKA
100 µm
RTKA-A
RTKA-NA
Supplemental Figure 2. Myofibroblast quantification of periarticular knee tissue. Myofibroblast quantification and representative immunofluorescence images of DAPI (blue), Laminin (red), α-SMA (green), and a merged image of all three stains from the three patient groups (PTKA=primary TKA, RTKA-A=arthrofibrosis, RTKA-NA=non-arthrofibrosis). The scale bar applies to all images in this figure.
