# Supplementary material for: Immune cell populations differ in patients undergoing revision total knee arthroplasty for arthrofibrosis
Source: Sci Rep. 2022 Dec 31;12:22627. doi: 10.1038/s41598-022-22175-3 (PMC9805429; doi:10.1038/s41598-022-22175-3)
Supplement: Supplementary file 2 — Supplementary Table 1. [file 41598_2022_22175_MOESM2_ESM.docx]

**Supplemental Table 1.** Information regarding the antibodies utilized.

| **Antibody Name** | **Antibody Used to Detect** | **Vendor** | **Catalog#** | **Clone** | **Type of Antibody** | **Epitope Retrieval/ time** | **Protein Block/ time** | **Primary Ab dilution/diluent** | **Primary Ab incubation time** | **Tissue Control** |
| --- | --- | --- | --- | --- | --- | --- | --- | --- | --- | --- |
| CD117 (c-Kit) | Mast Cells | Agilent (Dako) | A4502 | N/A | Rabbit Polyclonal | ER2 / 20min | Protein Block / 5min | 1:500 in Bond Diluent | 15min | Tonsil |
| CD163 | Macrophages | Leica Biosystems | CD163-L-CE | 10D6 | Mouse Monoclonal | ER2 / 20min | Protein Block / 5min | 1:400 in Background Reducing | 15min | Tonsil |
| CD20cy | B-cells | Agilent (Dako) | M0755 | L26 | Mouse Monoclonal | ER2 / 20min | None | 1:400 in Background Reducing | 15min | Tonsil |
| CD3 | T-cells | Agilent (Dako) | M7254 | F7.2.38 | Mouse Monoclonal | ER2 / 30min | None | 1:200 in Bond Diluent | 15min | Tonsil |
| Alpha-SMA | Myofibroblasts, Blood Vessels | Millipore Sigma | F3777 | 1A4 | Mouse monoclonal | None | Protein Block / 30min | 1:200 in Antibody Diluent | Overnight | Small Intestine |
| Laminin | Blood Vessels | R&D Systems | AF7340 | N/A | Sheep Polyclonal | None | Protein Block / 30min | 1:20 in Antibody Diluent | Overnight |  |
