# Supplementary material for: Immune cell populations differ in patients undergoing revision total knee arthroplasty for arthrofibrosis
Source: Sci Rep. 2022 Dec 31;12:22627. doi: 10.1038/s41598-022-22175-3 (PMC9805429; doi:10.1038/s41598-022-22175-3)
Supplement: Supplementary file 3 — Supplementary Table 2. [file 41598_2022_22175_MOESM3_ESM.docx]

**Supplemental Table 2.** Mean cell densities and effect size calculations presented as Cohen’s *d* values for CD163+ macrophages, CD117+ mast cells, CD3+ T-cells and CD20+ B-cells in the whole tissue and in the fibrous, adipose and synovial tissue regions.

Abbreviations: PTKA = primary TKA; RTKA-A = revision TKA for arthrofibrosis; RTKA-NA = revision TKA for non-arthrofibrotic reasons; SD = standard deviation

*Note: there was only one RTKA-NA sample with synovial tissue; unable to perform statistical analysis with only one sample

| **Marker** | **Region** | **PTKA Mean Cell Density (SD)** | **RTKA-A Mean Cell Density (SD)** | **RTKA-NA Mean Cell Density (SD)** | **Cohen’s *d* PTKA vs. RTKA-A** | **Cohen’s *d* RTKA-NA vs. RTKA-A** | **Cohen’s *d* PTKA vs. RTKA-NA** |
| --- | --- | --- | --- | --- | --- | --- | --- |
| **CD163** | Overall | 413.0 (298.9) | 251.7 (131.6) | 673.7 (938.0) | 0.698 | 0.717 | 0.355 |
|  | Fibrous | 474.3 (249.5) | 240.9 (140.8) | 598.3 (512.5) | 1.152 | 1.15 | 0.35 |
|  | Adipose | 222.9 (149.6) | 278.9 (142.6) | 104.4 (47.0) | 0.383 | 1.444 | 0.937 |
|  | Synovium | 3004.6 (1225.3) | 1796.4 (1273.1) | 3623.0 (0) | 0.967 | N/A* | N/A* |
| **CD117** | Overall | 13.2 (10.2) | 18.4 (19.8) | 10.9 (9.9) | 0.33 | 0.434 | 0.228 |
|  | Fibrous | 27.2 (31.4) | 21.7 (21.0) | 15.0 (12.0) | 0.206 | 0.36 | 0.455 |
|  | Adipose | 6.3 (4.7) | 12.3 (8.5) | 10.4 (11.9) | 0.874 | 0.196 | 0.53 |
|  | Synovium | 59.9 (66.9) | 3.6 (4.1) | 14.6 (0) | 1.188 | N/A* | N/A* |
| **CD3** | Overall | 2.7 (3.0) | 4.8 (5.5) | 9.3 (9.9) | 0.474 | 0.626 | 1.083 |
|  | Fibrous | 4.7 (6.1) | 3.7 (4.4) | 9.1 (9.8) | 0.188 | 0.818 | 0.589 |
|  | Adipose | 1.3 (1.7) | 6.5 (7.0) | 1.4 (2.0) | 1.021 | 0.865 | 0.056 |
|  | Synovium | 9.8 (12.9) | 0.4 (0.6) | 12.4 (0) | 1.029 | N/A* | N/A* |
| **CD20** | Overall | 6.8 (8.6) | 3.3 (5.0) | 7.9 (13.2) | 0.498 | 0.542 | 0.107 |
|  | Fibrous | 9.9 (11.3) | 4.0 (7.5) | 8.2 (13.1) | 0.615 | 0.436 | 0.143 |
|  | Adipose | 1.4 (1.2) | 3.5 (4.8) | 1.0 (1.8) | 0.6 | 0.61 | 0.282 |
|  | Synovium | 32.3 (58.1) | 0.95 (1.3) | 6.6 (0) | 0.763 | N/A* | N/A* |
